# Detection of blood aspiration in deadly head gunshots comparing postmortem computed tomography (PMCT) and autopsy

**DOI:** 10.1186/s40001-016-0237-6

**Published:** 2016-11-01

**Authors:** E. Scaparra, O. Peschel, C. Kirchhoff, M. Reiser, S. M. Kirchhoff

**Affiliations:** 1 Ludwig-Maximilians-University Munich, Munich, Germany; 2 Institute of Forensic Medicine, Ludwig Maximilians University, Nussbaumstrasse 11, 80336 Munich, Germany; 3 Department of Trauma Surgery, Klinikum Rechts der Isar, Technische Universität München, Ismaninger Strasse 22, 81675 Munich, Germany; 4 Institute of Clinical Radiology, Ludwig Maximilians University Munich, Klinikum Großhadern, Marchioninistrasse 15, 81377 Munich, Germany

**Keywords:** Gunshot, Head, Blood aspiration, Postmortem computed tomography, Autopsy, Comparison

## Abstract

**Background:**

The aim of our study was to analyze the reliability of postmortem computed tomography (PMCT) versus autopsy in detecting signs of blood aspiration in a distinct group of patients following deadly head, mouth or floor of mouth gunshot injuries.

**Methods:**

In this study, in 41 cases PMCT was compared to autopsy reports, the gold standard of postmortem exams, regarding detection of blood aspiration. PMCT was evaluated for the presence and level of typical signs of blood aspiration in the major airways and lung using a semi-quantitative scale ranging from level 0 (no aspiration) to 3 (significant aspiration) also taking density values of the described potential aspiratory changes into account.

**Results:**

Overall, in 29 (70.7%) of 41 enrolled cases PMCT and autopsy revealed the same level of aspiration. A difference of one level between PMCT and autopsy resulted for 5 (12.2%) of the remaining 12 cases. More than one level difference between both methods resulted for 7 cases (17.2%). Autopsy described no signs of aspiration in 10 cases, compared to 31 cases with reported blood aspiration. In contrast, PMCT revealed no signs of blood aspiration in 15 cases whereas 26 cases were rated as positive for signs of aspiration in the major airways. In 18 of these 26 cases considered positive for blood aspiration by autopsy and PMCT, *clear* signs of aspiration signs were also described bilaterally by both methods.

**Conclusions:**

The presented study provides evidence for the assumption that PMCT seems to be helpful in the detection of blood aspiration in cases of deadly head gunshots. In conclusion, it seems reasonable to suggest performing PMCT additionally to traditional postmortem exams in cases of suspected aspiration to rule out false-negative cases and to possibly allow for a more detailed and rather evidence based examination reconnoitering the cause of death. However, the adequate use of PMCT in this context needs further evaluation and the definition of an objective scale for aspiration detection on PMCT needs to be established in future studies.

## Background

One of the major issues in forensic practice is to determine whether an injury was inflicted during life or after death [1, 2]. The reliability of the four basic vital reactions needs to be carefully assessed, since several confounders, e.g., petechial bleeding caused by hypostasis, potentially may alter the final aspect [3, 4].

Breathing is exclusively triggered by the central nervous system depending on its activity. Thus, if breathing stops along with circulatory arrest or brain death signs of vitality based on an intact respiration can highly reliable be attributed [3, 4]. Therefore, one of the major pulmonary vital reactions is aspiration. Detecting signs of aspiration is of high forensic relevance due to the fact that it provides information whether an injury occurred pre- or postmortem and/or it was the primary or contributing factor to the cause of death. Especially blood aspiration with blood found deep in the bronchial tree is accepted as a sign of vitality. However, of course blood resulting from, e.g., resuscitation maneuvers, etc., perimortal and even postmortem flow of material into the respiratory tract should be excluded first in this context [5].

The typical macroscopic appearance of blood aspiration during forensic autopsy comprises red rounded areas on the surface of the lungs and on the cut surfaces. These macroscopic findings are eventually confirmed by the microscopic description of small airways filled with blood. In addition also the major airways are examined for the presence of intraluminal blood [2–4].

While autopsy and its traditional subspecialties like histopathology represent the current gold standard for differentiation of pre- and postmortem injuries, postmortem computed tomography (PMCT) is gaining more and more relevance in forensic medicine [3–7]. In ballistic analysis, PMCT presents a non-invasive, but rather effective imaging technique especially for reconstruction of projectile tracks [8–12]. However, of course besides advantages in ballistic analysis, PMCT offers further positive characteristics compared to autopsy in terms of detection of intracorporeal gas, detection of fractures and/or foreign bodies.

Recently, Filograna et al. provided first evidence of the applicability of PMCT in detection of blood aspiration in a gross series of death for different reasons [13]. Therefore, the aim of our study was to analyze the reliability of PMCT versus autopsy in detecting signs of blood aspiration in a distinct group of patients following deadly head, mouth or floor of mouth gunshot injuries.

## Methods

### Subjects

The study protocol was approved by the University’s board of ethics (Reference nr. 151/08). For the presented retrospective study, all whole PMCTs with the suspicion for deadly head gunshot including gunshots to the mouth and floor of mouth between October 2008 and April 2011 were enrolled. All cases with additional chest trauma were excluded afterwards to rule out retrograde blood aspiration [14]. Moreover, all cases with any kind of resuscitation maneuvers documented or suspected were excluded.

According to our study protocol, in a first step PMCT was performed followed by autopsy and forensic analysis in a second step. Date, potential cause of death and position after death were found by the reading radiologist during image analysis. All autopsy findings were blinded to the radiologist and PMCT findings were blinded to the forensic pathologists. In a final step, PMCT and autopsy analysis were compared for signs and extent of blood aspiration.

### Postmortem computed tomography (PMCT) imaging and analysis

PMCT was performed in a standardized manner with the corpses lying in a supine position. All corpses were kept within the body bag they were deposited after finding on scene. A native CT scan, i.e., without administration of contrast agent was performed on a 64-slice scanner (Brilliance 64 Philips, Amsterdam, Netherlands; GE Discovery 750 HD GE Healthcare Massachusetts USA). First, a scan scout of the head and cervical spine was performed followed by the CT itself with axial reformats in 3.75 mm thickness. The CT scan of the thoracic and abdominal cavity including pelvis and parts of the lower extremities up to a maximum scan length of 200 cm (GE), respectively, 180 cm (Philips) was reformatted in 1.25-mm axial slices. Following scan procedures, PMCT data were transferred to the PACS (picture achieving computer system) for storage and further evaluation.

PMCT data were read and evaluated by one board-certified radiologist with expert experience in forensic radiology and one radiology resident with novice experience. Findings were stated in a consensus reading of both.

PMCT analysis comprised the evaluation of the airways as well as of the lung. Criteria for blood assessment in the trachea were on the one hand observing sedimentation and/or potential luminal airway occlusion and on the other hand measuring Hounsfield Units (HU) for the presence of blood-like density values with a range from 20 to 90 HU being considered since blood can potentially sediment and exhibit different blood typically HU compared to the lung. Signs of aspiration within the major airways were classified using a four-level scale (see Table 1).

**Table 1:** Grading system for findings suggestive for aspiration within the major airways

Level	Grade	Major airways	
		PMCT morphology	Autopsy
0	None	No signs of aspiration	No signs of aspiration
I	Minimal	Slight blood deposits at the wall of the airways	Slight blood deposits at the wall of airways
II	Moderate	Significant blood deposits at the walls of the major airways	Significant blood deposits at the walls of major airways
III	Significant	Maximum signs of aspiration with complete obstruction of the lumen of the airways	Maximum signs of aspiration with complete obstruction of the lumen of airways

The scale ranges from “none” to “significant” whereas none means level 0 and significant level III. Descriptive findings for each, PMCT as well as autopsy are given

**Fig. 1:**
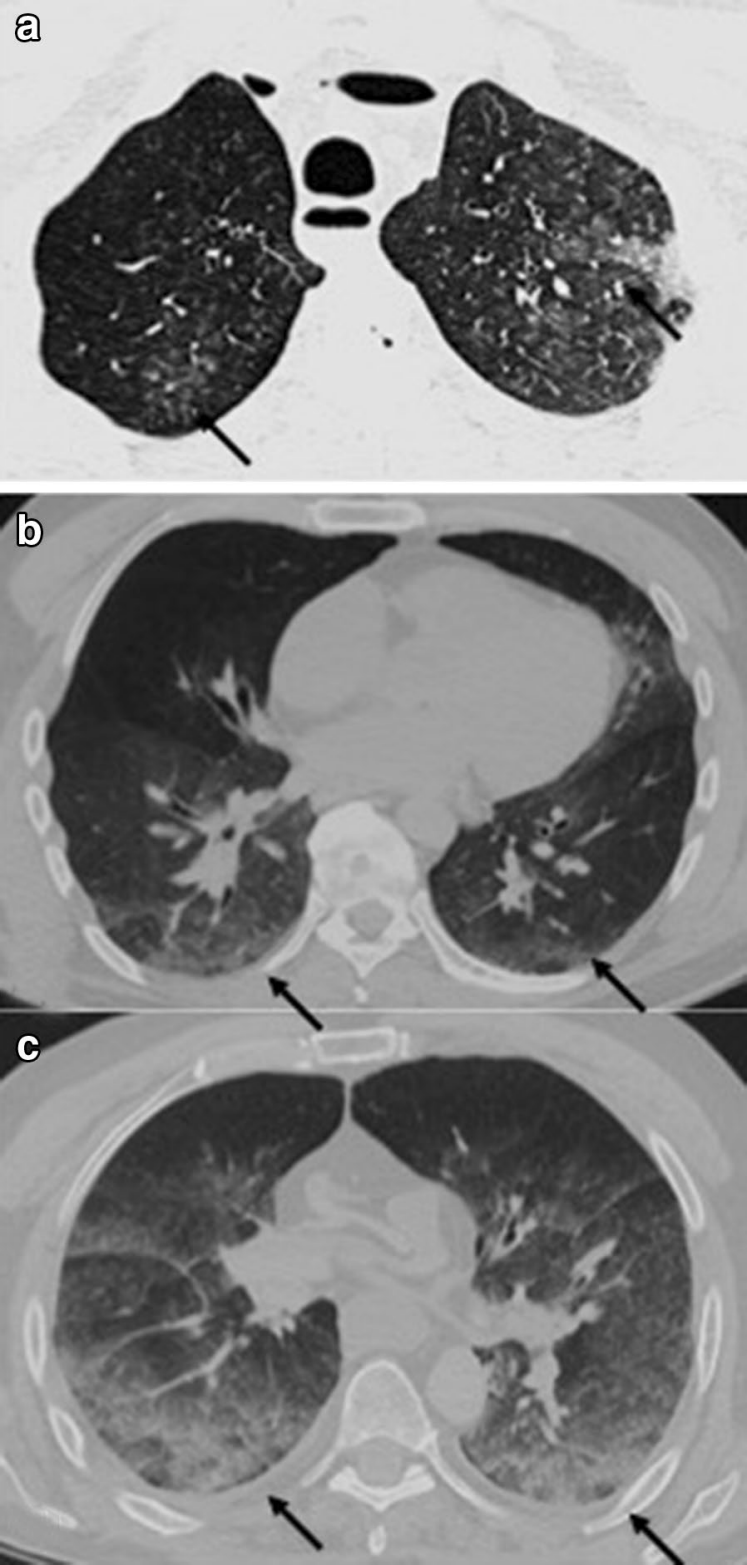
**a**–**c** The grading scale of blood aspiration. (**a**) Grade I showing only a few ground glass opacities (*arrows*) without any signs of confluence. (**b**) Grade II in terms of increasing number of ground glass opacities (*arrows*) with discrete signs of confluence. **c** Grade III of blood aspiration on PMCT in terms of multiple ggos with distinct signs of confluence

Findings such as round, possibly converging spots with irregular margins and ground glass opacities (ggo) in the lung parenchyma with blood-like density values of 50–70 Hounsfield units (HU) were considered as suggestive for blood aspiration on PMCT [15]. The level of blood aspiration within the lung parenchyma was defined according to Table 2 (see Fig. 1).

**Table 2:** Grading system for findings suggestive for aspiration within the minor airways as well as the lung

Level	Grade	Minor airways/lung parenchyma	
		PMCT morphology	Autopsy
0	None	No ground glass opacities (ggo)	No signs of aspiration
I	Minimal	Few ggos without signs of confluence	Slight blood deposits at the wall of the lungs
II	Moderate	Many ggos, partial confluence	Significant blood deposits at the walls of the lungs
III	Significant	Many ggos, great confluence	Maximum signs of aspiration with complete obstruction of the lumen of the lungs

The scale ranges from “none” to “significant” whereas none means level 0 and significant level III. Descriptive findings for each, PMCT as well as autopsy are given

### Autopsy and analysis

Following PMCT, the corpses underwent autopsy at our University Institute of Forensic medicine. Board-certified forensic pathologists performed all autopsies according to the standards of the German Government’s guidelines (§87, 89 German Code of Criminal Procedure). Standard autopsy in lethal ballistic injury comprises first the inspection of the entire skin looking for the entrance and exit wound, respectively, second by opening of all three body cavities (skull, thorax and abdomen), and third the dedicated examination of all internal organs. The respiratory tract was resected en block by transecting the trachea right below the glottis. In the following, the airways and pulmonary blood vessels were inspected and dissected. Then the lung surface was inspected and the lung parenchyma was cut into 1.5-cm slices of varying thickness to assess for potential signs of aspiration from the apex to the base. Tissue samples from all organs were harvested for histopathology.

Autopsy reports were reviewed for the description of blood or blood-like fluid in the larynx, trachea, main bronchi considered as major airways and small bronchi but also for the description of red rounded spots with irregular margin and diffuse pattern on the lungs’ surface or in the cutting area. Parallel to the PMCT evaluation, aspiration within the major airways was classified using the four-level scale and the degree of blood aspiration within the lung parenchyma according to Tables 1 and 2. Diagnosis of blood aspiration of the lung was confirmed by histology.

## Results

### General data

Between October 2008 and April 2011, overall 57 cases with head gunshot-related death underwent PMCT and autopsy. 16 head shot cases sustained additional gunshot to chest and/or thoracic spine so that they were excluded from the study.

Consecutively, 41 PMCTs of 7 women (17.1%) and 34 men (82.9%) with a median age at death of 57 years (range: 14–89 years) were enrolled. PMCT and autopsy were performed at a median of 26 h (range 12–30 h) and 50 h (30–56 h) after death, respectively. The pattern of gunshots comprised 20 gunshots to the head (48.8%), four gunshots through the floor of mouth (9.8%) and 17 gunshots into the mouth (41.5%) (see Table 3).

**Table 3:** General patients’ characteristics

PMCT enrolled	34 male (82.9%)	7 female (17.1%)	
Age range (years)	14–89	Median 57	
Gunshot pattern	20 head (48.8%)	4 floor of mouth (9.8%)	17 mouth (41.5%)

### Major airways

The status of the airways regarding the present signs of aspiration was rated as equal in autopsy and PMCT in 22 of the 41 enrolled cases. In 11 cases, a gradual difference was found, whereas in 8 cases a total difference resulted.

In autopsy, in 10 cases no signs of blood aspiration in the airways were described, whereas 31 cases were reported with blood in the airways.

PMCT revealed 26 cases with blood equal density material in the airways and 15 cases without signs of blood aspiration in the major airways (see Table 4).

**Table 4:** Results for comparison of PMCT and autopsy

	No diff	1-level diff	2-level diff	3-level diff
PMCT > Autopsy		3 (7.3%)	1 (2.4%)	2 (4.9%)
Autopsy > PMCT		2 (4.9%)	1(2.4%)	3 (7.3%)
Total	29 (70.7%)	5 (12.2%)	2 (4.9%)	5 (12.2%)

### Lungs

In 25 cases, blood-like content was described by both, PMCT and autopsy, (Fig. 1) within the major airways. In 18 of these 25 cases, significant signs of aspiration were detected in the lungs as well by PMCT and autopsy.

### PMCT versus autopsy

**Fig. 2:**
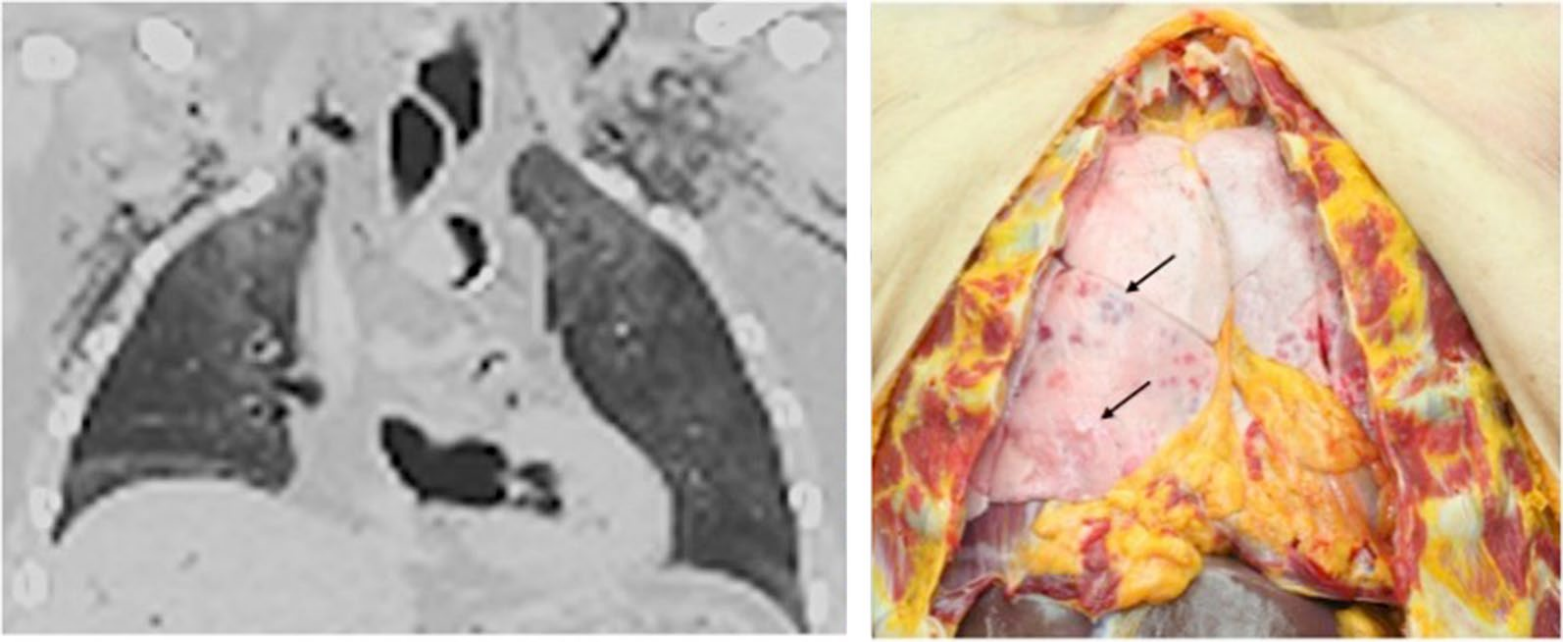
The *left* coronal reformat of pmCT demonstrates grade I of blood aspiration with the correlate in the gold standard autopsy showing only a few *red rounded* spots with irregular margin and diffuse pattern on the lungs’ surface (*arrows*)

**Fig. 3:**
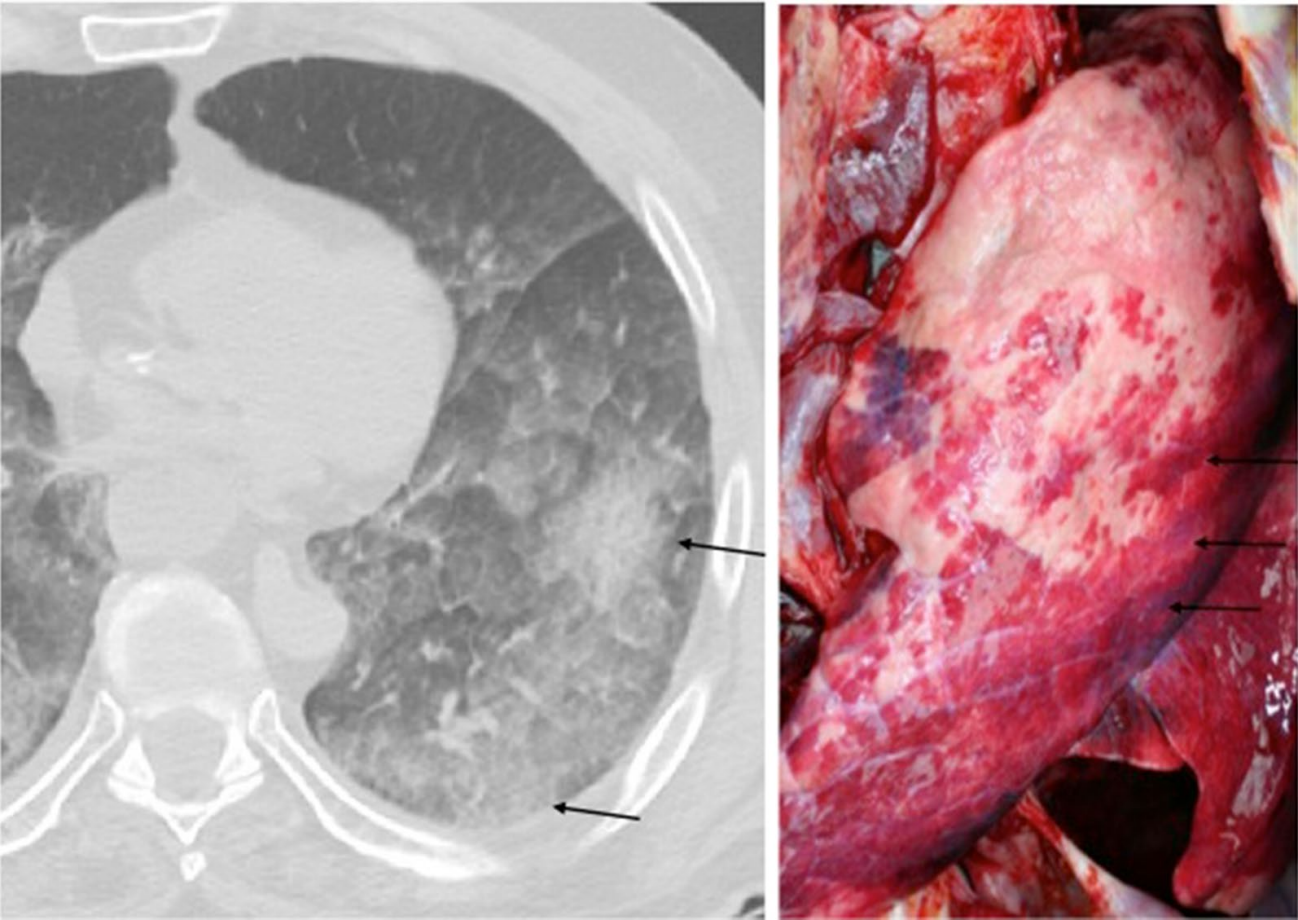
Grade III of blood aspiration on PMCT (*left image*, axial PMCT-reformat) presenting multiple ground glass opacities with significant confluence (*arrows*) and the corresponding morphological correlate in autopsy showing multiple *red rounded* spots with irregular margin, diffuse pattern on the lungs’ surface with significant confluence (*arrows*)

**Fig. 4:**
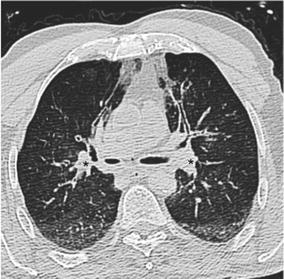
The case demonstrates occlusion by blood dense material of the major airways (*star*) without signs of blood aspiration in the lungs by axial PMCT-reformat

29 (70.7%) of the enrolled 41 cases presented equal signs of aspiration on PMCT and autopsy. In 6/29 cases no signs of aspiration (level 0) were reported. In one case, level I aspiration was assessed (see Fig. 2), in two cases level 2 and in 18 cases aspiration level 3 (see Figs. 3, 4) resulted.

In 5 cases (12.2%), 1° difference on PMCT than stated in the autopsy reports was found. 7 cases (17.1%) were evaluated with more than one level difference between gold standard and PMCT. 5 (12.2%) of these 7 cases were graded with a difference of three levels between autopsy and PMCT and 2 (4.9%) with two-level difference. In these cases, the autopsy reports described no signs of aspiration whereas PMCT described level 2 aspiration (A: 0 PMCT: 2). Two cases were graded in autopsy with level 0 and in PMCT were (Fig. 4) graded as level 3 (A: 0 PMCT: 3). Three cases were graded in autopsy with level 3, whereas in PMCT was evaluated as level 0 (see Table 4).

## Discussion

In the current literature, the use of postmortem CT in relation to the gold standard autopsy is constantly discussed [3, 8]. However, to the best of our knowledge, there exists no study about the potential of PMCT for the detection of blood aspiration in deadly gunshot injuries to the head, to the mouth or mouth floor in comparison to the gold standard autopsy. The actual study showed in 29 of 41 enrolled cases the same location and extent of blood aspiration in the major airways and in the lungs on PMCT and in conventional autopsy. In addition, the evaluation of the status of the major airways and lung revealed almost identical results comparing PMCT to autopsy. Thus, the presented results provide evidence that PMCT might potentially help in the detection of blood aspiration in cases of deadly head gunshot cases. Breathing may terminate along with circulatory arrest and/or brain death so that the detection of blood aspiration can support the forensic pathologists regarding the assessment whether an injury had occurred pre-, peri- or postmortem. In addition, suction of great amount of blood into the deep respiratory tract might help in determining the cause of death.

### PMCT in general

In general, modern cross-sectional imaging techniques are already considered as reliable exams complementary to conventional forensic techniques especially autopsy [16, 17]. In the context of gunshot-related death, PMCT presents a non-invasive, but effective imaging technique to localize gunshot wound tracks and support findings of autopsy in gunshot victims [16–20]. Furthermore, PMCT is useful in traumatic death allowing for an immediate identification of causes of death providing detailed information on bony lesions, brain injuries and gas formations [21].

### PMCT and blood aspiration

Reviewing the current literature, detection of blood aspiration by PMCT has only been discussed by Filograna et al. [13, 22]. They reported cases on a retrospective basis regarding signs of aspiration in autopsy and afterwards PMCT data were retrospectively analyzed for signs of blood aspiration. In general, the results of these studies are comparable to our results regarding the detection rates of aspiration on PMCT. Therefore, in the presented study, a rather prospective way of assessing signs of blood aspiration on PMCT was chosen and compared to the autopsy as gold standard. All of our cases have not been preselected in terms of aspiration signs to evaluate the pure detection rates of aspiration on PMCT.

In the presented study, a semi-quantitative scale to rate blood aspiration was used. Other authors used a scale differentiating between quantity, position and consolidation of ground glass opacities and the presence of damage to the lungs using an evaluation of differentiating between yes/no, scarce and many ground glass opacities and consolidations [13, 22]. To simplify the evaluation procedure, we used a scaling which was geared to the autopsy reports meaning that PMCT findings were graded in degrees based on the quantity and characteristics of ground glass opacities (ggo) inside the lungs and on the content of the major airways. Every semi-quantitative scaling is affected with the problem that it depends on the interpreter and his experience in evaluating such data. Thus, the availability of objective scales would be of great value for the PMCT aspiration detection. In this context, one potential option might be the definition of CT images with signs of aspirations and accounting the aspiration spots, measuring the density in HU and evaluating the confluence of these opacities [23].

Regarding the results of the presented study for 70% of the included cases, the same degree of aspiration in PMCT and autopsy resulted. Five cases showed only 1° difference between both methods whereas five cases had a difference of three degrees meaning that over 82% of aspiration was shown by PMCT. In 3 of these 5 cases, autopsy described severe signs of aspiration compared to PMCT findings and in the remaining 2 cases PMCT showed more signs of aspiration than the gold standard. Possible reasons for these grading differences might be that other pathologic findings mimic ggos as for PMCT in terms of beginning lung edema or posttraumatic changes and thus were misinterpreted as signs of aspiration. In three of these five cases, the differentiation between clouded spots in PMCT and ggos due to aspiration was nearly impossible. This might be due to sedimentation or decomposing processes in the base of the lungs. Another reason for autopsy rating aspiration levels higher than PMCT might be that it was not adequately assessed and reported in autopsy. Autopsy reports were evaluated retrospectively whereas the PMCT data sets were evaluated prospectively, so there was no possibility to reevaluate the findings in autopsy. Also on PMCT, findings such as gastric content might mimic ggos thus might explain some cases of misinterpretation. However, measuring the density of changes of the lung parenchyma provides some help in differentiating blood from, e.g., gastric content.

Since in the current literature to the best of our knowledge no study with a comparable study design exists, a comparison to the actual literature is rather difficult. Overall reviewing the subject of our study, it is safe to say that PMCT can help to find and quantify aspiration in cases of gunshot-related death to the head, mouth or floor of mouth. To confirm PMCT as a standard procedure for aspiration detection in forensic cases, it is necessary to further investigate and evaluate an objective scale to set standards and define the limits and advantages of PMCT in this matter [24].

In this context, one needs to be aware of the fact that blood possibly reaches the airways not only in an antegrade way, for example following traumatic skull base fractures, but also in a retrograde way, e.g., following injury to lung parenchyma beneath the bronchial tree. However, it should also be mentioned that such retrograde aspiration is far less present than antegrade “typical” aspiration and is usually in close relation regarding location to its origin in terms of lung injury [14].

### Strength and limitations of PMCT

In general, PMCT presents a non-invasive valuable imaging technique with only a few decades of experience whereas autopsy owns hundreds of years of experience as well as an unlimited field of examination. In this context, it should be mentioned that several body areas, e.g., maxilla-facial region, are very hard to access in autopsy and often in only a destructive way so that pathologies in these regions are only examined in autopsy if indirect signs are present which is easily examined and also evaluated by PMCT [25]. Another strength of PMCT is the fact that the exam itself is performed faster than autopsy [26]. In addition, the original CT data are documented, stored in the archives and reevaluated if necessary. Another limitation of PMCT in general is the high expenses for purchasing a CT scanner. In addition, the expenses for a whole body PMCT is relatively high compared to the expenses regarding autopsy although the information depriving from PMCT is great especially in context with the detection of blood and aspiration helping to a great extent in solving the puzzle of the injury happening pre-, peri- or postmortem [8, 27]. Regarding the presented study one strength of PMCT is the fact that PMCT easily provides a visualization of the entire lung parenchyma with the consequence of avoiding an underestimation of pulmonary aspiration in autopsy. However, the number of enrolled cases is relatively low so that a prospective study is recommended to confirm the results of our imaging analysis on a larger patient cohort.

## Conclusions

In conclusion, it seems reasonable to suggest performing PMCT additionally to traditional postmortem exams in terms of autopsy in cases of suspected blood aspiration to rule out false-negative cases since PMCT of the lungs and airways alone does not provide enough information to certainly differentiate between findings of the lung following blood aspiration and due to other causes. CT provides an easy visualization of the entire lung parenchyma with the possibility to avoid underestimation of the severity of aspiration in autopsy. Thus, CT imaging should be considered as complementary tool to conventional autopsy assessing blood aspiration as a sign of vital reaction or the cause of death. However, the adequate use of PMCT in this context needs further evaluation and the definition of an objective scale for aspiration detection on PMCT needs to be established in future studies.

## Abbreviations

PMCT: postmortem computed tomography
CT: computed tomography
HU: Hounsfield units
Ggo: ground glass opacity
